# Parental HIV/AIDS status and death, and children's psychological wellbeing

**DOI:** 10.1186/1752-4458-3-26

**Published:** 2009-11-24

**Authors:** Paul Narh Doku

**Affiliations:** 1 Community Based Sciences, Section of Psychological Medicine, University of Glasgow, UK; 2 Institute of Psychology, Norwegian University of Science and Technology, Trondheim, Norway; 3 Regent University College of Science and Technology, Department of Human Development and Psychology, Ghana

## Abstract

**Background:**

Ghana has an estimated one million orphans, 250,000 are due to AIDS parental deaths. This is the first study that examined the impact of parental HIV/AIDS status and death on the mental health of children in Ghana.

**Methods:**

In a cross-sectional survey, 4 groups of 200 children (children whose parents died of AIDS, children whose parents died of causes other than AIDS, children living with parents infected with HIV/AIDS, and non-orphaned children whose parents are not known to be infected with HIV/AIDS) aged between 10 and 19 were interviewed on their hyperactivity, emotional, conduct, and peer problems using the Strengths and Difficulties Questionnaire.

**Results:**

Children whose parents died of AIDS showed very high levels of peer problems [F (3,196) = 7.34, p < .001] whilst both orphaned groups scored similarly high on conduct problems [F (3, 196) = 14.85, p < .001]. Hyperactivity showed no difference and was very low in the entire sample. Emotional problems were very high in all the groups except among the non-orphaned children [F (3, 196) = 5.10, p < .001].

**Conclusion:**

Orphans and children living with parents infected with HIV/AIDS are at heightened risks for emotional and behavioural disorders and that efforts to address problems in children affected by HIV/AIDS must focus on both groups of children. Parallel to this, researchers should see these findings as generated hypotheses (rather than conclusions) calling for further exploration of specific causal linkages between HIV/AIDS and children's mental health, using more rigorous research tools and designs.

## Background

One of the biggest challenges of present health care efforts in Africa in the area of HIV/AIDS is how to provide care and support for the soaring numbers of orphaned children being created by the disease. Over 14 million of African children are orphaned by HIV/AIDS deaths and it is projected that 20 million children will be orphaned by 2010; at which time 12% of all children in sub-Sahara Africa will be orphaned to AIDS [1]. Clearly, the African continent is in an "orphan crises" and lack of stable care is likely to put millions of African orphaned children at heightened risk for both physical and mental health problems.

It was suggested that the death of a parent, regardless of its cause could place children at risk for internalizing problems such as depression, anxiety, withdrawal, and low self-esteem [2]. The expressions of externalizing problems among orphaned children are much less consistent. AIDS orphaned children might not only be traumatized by the loss of parents (whose physical deterioration they may often have witnessed), they may also lack the necessary parental guidance through crucial life-stages of identity formation and socialization into adulthood amidst AIDS related stigma and discrimination. Yet much effort is still being devoted (by African governments) to counting orphans and too little being done to identify broader risks to their health and development.

### Earlier Studies in Africa

Despite these shortcomings, a few studies have examined the mental health needs of these children. A researcher used children's and parents' scores from both the Rand and Beck Inventories to compare 76 orphaned and 74 non-orphaned children on their psychological wellbeing [3]. The author found that in Mozambique, orphans have higher depression symptoms, were easily bullied and less likely to have trusted friends than non-orphans. In a related study, 41 each of orphans and non-orphans were interviewed using an adapted version of the Rand Mental Health and Beck Depression Inventories translated into Swahili [4]. The translated measure with a reliability alpha of 0.83 revealed that orphaned children showed markedly higher internalizing problems, increased suicidal ideation, often went to bed hungry and were more likely to be out of school. The researchers reported recruiting the children with community help, local leaders and some NGOs already doing some care and support interventions in the community. The problem here is that the study suffers from selection bias that might have influenced the findings. Another issue is that several studies consistently show the strong correlation between poor family socioeconomic status and psychological problems [5-7]. The researchers showed that significant numbers of orphaned children compared with non-orphaned children reported a lack of food at home, lack of money for school fees, books and uniform, and often going to bed hungry, suggesting a plausible socioeconomic explanation for any observed internalizing problems among the orphans.

In Uganda children whose parents died of AIDS were found to score significantly higher on anxiety, depression and aggression than non-orphaned children [8]. The researchers compared 123 children whose parents died of AIDS and 110 non-orphaned children using the Beck Youth Inventories translated into Runyankore (Ugandan dialect). In contrast, a related study that interviewed 30 children whose parents died of AIDS and 30 non-orphaned children in South Africa using the Strengths and Difficulties Questionnaire found no such significant differences between the two groups [9]. However, in the earlier study, individual BYI analyses revealed that items particularly sensitive to depression (hopelessness and suicidal ideation) were significantly higher for orphaned children [8]. Similarly, individual item analyses in the latter also showed some significant differences. Orphaned children were less likely to have a good friend, more likely to lose their temper quickly, more likely to exhibit difficulty concentrating and have somatic symptoms and nightmares [9]. In the two studies, both authors compared children whose parents died of AIDS and non-orphaned children on their psychological wellbeing and concluded that the differences between the groups were due to "AIDS orphanhood". Clearly, this line of logic is problematic as their choices of controlled (non-orphaned children) make it difficult to separate the impacts of "orphanhood" from "AIDS orphanhood". In fact, any such differences noted could equally be attributable to "orphanhood" and not "AIDS orphanhood" that the authors of both papers suggested.

More recently, two studies conducted in South Africa reported conflicting findings. The first study assessed psychological wellbeing among 81 children whose parents died of AIDS compared with 78 other orphaned and 43 non-orphaned children recruited through NGOs [10]. Findings from the study revealed that other orphaned children exhibited higher depression and anxiety symptoms than non-orphaned children with children whose parents died of AIDS showing intermittent scores. Whilst no significant differences were found for antisocial behaviours (externalizing problems), the Self-Esteem Questionnaire scores indicated that other orphaned children have lower self esteem than both non-orphaned and children whose parents died of AIDS. In the other study, 1025 children (425 orphaned by AIDS, 241 orphaned by other causes and 278 non-orphaned children) were interviewed [11]. The study used several measures including the Child Depression Inventory (short form), R- CMAS, SDQ, Child PTSD Checklist, and CBCL-YSR. The main finding reported by the researchers was that orphanhood by AIDS is significantly associated with depression (after age and sex were adjusted), peer problems, post-traumatic stress, delinquency and conduct problems, but only marginal with anxiety. Orphanhood by other causes was moderately associated with post-traumatic stress in an unadjusted model. The researchers also reported the that more AIDS orphaned children than non-orphaned children met clinical disorder cut-offs points for depression, anxiety, peer problems, post-traumatic stress, delinquency, and conduct problems.

### Conclusion and aims of the study

Overall, the findings of the literature on children whose parents died of AIDS suggest higher internalizing problems and some externalizing problems among orphans. However, the evidence for these observations is not only limited, grey and scattered but also contradictory and often controversial considering their weak methodological designs and choices of control groups noted above. Therefore, the question as to whether children orphaned by AIDS have unique needs and problems different from children orphaned by other causes is only partially answered, if answered at all. Further research is therefore necessary in this area. Researchers cautioned the danger of assuming that studies conducted in one part of Africa are transferable to other African contexts [11], and this study is the first quantitative study to examine the mental health of these vulnerable children in Ghana.

Recognizing that the impact of HIV/AIDS on children may start far before they are orphaned, the present study goes further to include children living with parents who are infected with HIV/AIDS [12-18]. To gain a better understanding about how children are affected by the HIV/AIDS pandemic research should compare groups of children whose parents died of AIDS, children whose parents died of causes other than AIDS, children living with parents infected with HIV/AIDS, and non-orphaned children whose parents are not known to be infected with HIV/AIDS in a single design to test the postulations reported by earlier researches. To date the present study is the first to do this. In this study, "***an orphan***" refers to a child below 18 years who lost one or both parents, "***other orphaned children***" refer to children whose parent(s) died of causes other than AIDS, and a "***non-orphan children***" are children living with parents not diagnosed with HIV/AIDS. Similarly, "***children whose parents died of AIDS***" refer to a child who has one or both parent died of AIDS.

## Methods

### Participants

The participants for this study were children aged 10 to 18 recruited from poor socioeconomic background throughout the Manya Krobo district of Ghana. Two hundred and seventeen were approached in their households and 200, (representing 92.2% response rate), participated. There were 97 males and 103 females. In this cross-sectional comparative cohort study 50 children orphaned by AIDS, 51 children orphaned through other causes, 48 children living with parents infected with HIV/AIDS, and 51 non-orphaned children were recruited. The children were recruited through community household visits. However, a few of those living with parents who have HIV/AIDS were identified with the help of the Manya Krobo Queen Mothers Association who happened to know their residences or households. The Queen Mothers Association is a group of 371 Queen Mothers across the six divisions of the Manya Krobo Traditional Area in the Eastern Region of Ghana. These female chiefs are responsible for the welfare of women and children under the communities they control. As part of their HIV/AIDS prevention and care programme, the Queen Mothers provide support and education to rural women, orphans and vulnerable children in the district of Manya Krobo. Currently, they oversee the welfare and education of over 1000 AIDS orphans in the area. Finally, some non-orphaned children and children made orphans by causes other than AIDS were also recruited through school visits. A child who was not orphaned but living with other relatives or caregivers other than their parents was excluded from the study. The study was conducted as a community household survey and so neither Home/Orphanage placed children were included nor the HIV status of children identified. In the case of parental HIV/AIDS status and death, verbal autopsy, a well validated method of cause of death identification in Ghana and some other African countries was used [11,19,20]. In this regard, a short inclusion criteria checklist with questions on cause of death or terminal illness, place and certification of death (if any), major signs and symptoms of HIV/AIDS were asked.

### Measure

A well validated measure, the Strengths and Difficulties Questionnaire (SDQ) [21], already translated into over 60 languages and used in 40 countries was used to assess children's psychological outcomes. The SDQ asks 25 items rated on a three-point likert scale (Not True, Somewhat True, and Certainly True) divided on five subscales: emotional symptoms, conduct problems, hyperactivity, peer relationship problems and prosocial behaviours. The sum of the first four subscales gives the total psychological difficulties per child. Each subscale of the SDQ has five items. The study utilized only the self completion version. Although the SDQ is not validated for the Ghanaian culture, it is used in this study based on the fact the measure correlates well with other measures such as the Child Behaviour Checklist, the Rutter Child Scales and the Child and Adolescent Burden Assessment that are all validated for Ghana [21,22]. Secondly, the SDQ is a measure well established for its power to discriminate well between community-based samples of children and adolescents [21]. Finally, the use of the SDQ does not only permit fast assessment of the participants' psychological health that facilitates screening and identification of at-risk children, it is also cost-effective. The study was conducted in English.

### Procedure

Households in Odumase Township were approached to take part in the study. Series of meetings were arranged with the council of elders and opinion leaders in the township to discuss the details of the study. Starting from a purposefully selected house at a cross-road (Odumase junction), every twentieth house is approached at cardinal directions and the inclusion checklist (parental status, the age and English fluency of the child) assessed. First, the nature and purpose of the study were explained to both potential child and parent or caregiver (and practically every adult present). When both the child and parent or caregiver consented and signed the consent forms to take part in the study, then the researcher and the child moved away to a quiet place to maintain confidentiality and privacy of the data collection process. Thus, consent was signed where inclusion criteria were met, and the data collected. With a copy of the SDQ given to the child, the items were then read aloud and the child ticked their responses (Not True, Somewhat True, and Certainly True). Although the interview was conducted in English, some necessary clarifications were made in the child's local dialect. At the end of the interview each child was thanked and offered the chance to ask questions about the research. A few children were interviewed in their schools where consent was obtained from both the children and their teachers.

### Ethics

HIV/AIDS issues are very sensitive topics and more so that the study involved children, considered to be vulnerable. Every effort was made to ensure that the families felt good about taking part in the study. Informed consent was obtained from both parents or caregiver and children before the study after the nature and purpose of the study, and data handling principles were thoroughly explained to them. They were made to understand that their children can withdraw from the study at any time even after consenting. The data collected was anonymous and the study was conducted in full accordance with the Helsinki Declaration of the World Medical Association. The research protocol and procedures were reviewed and approved by the Norwegian Regional Ethics Committee for Medical Research, Trondheim branch as well as the Department of Psychology, University of Ghana.

## Results

The mean age of participated children was 14.18 years with a standard deviation of 2.10. On the whole, there was no differences among the groups on age [F (3,196) = 0.793; p = n. s.]. There was also no significant difference between the ages of boys and girls (t = 1.859; p = n. s.). Using SPSS 15.0 a one Way MANOVA was conducted comparing the four groups (children whose parents died of AIDS, children whose parents died of causes other than AIDS, children living with parents infected with HIV/AIDS, and non-orphaned children whose parents are not known to be infected with HIV/AIDS) on their psychological outcomes with a .05 alpha level selected. In the analysis exposure of children to their parental AIDS status (groups) were entered as the independent variables and the psychological outcomes as dependent variables. Results indicated that parental HIV/AIDS status was significantly related to overall psychological outcome [F (5, 196) = 2.42, p < .001; Wilks Lambda = 0. 77]. A follow-up univariate analyses performed showed that HIV/AIDS exposure or status (group) was significantly associated with emotional problems [F (3, 196) = 5.10, p < .001], conduct problems [F (3, 196) = 14.85, p < .001] and peer problems [F (3,196) = 7.34, p < .001] but not hyperactivity [F (3, 196) = 0.51, p = n. s.] and prosocial behaviours [F (3, 196) = 0.28, p = n. s.]. These findings are presented in Table 1 along with summarized post hoc analyses.

**Table 1:** Means (M), standard deviations (SD) and MANOVA results of the 4 different groups on the SDQ subscales

	AIDS Orphans (A)		Other Orphans (B)		Children with AIDS Parents (C)		Non-orphans with Normal Parents (D)		Post hoc comparisons (Bonferroni test)		
							
Source	M	SD	M	SD	M	SD	M	SD	F(3, 196)	P	
Total Difficulties	20.08	4.37	17.92	4.50	16.38	5.20	14.67	5.20	11.34	0.001	**A > B = C = D**
Emotional Problems	6.84	1.39	6.61	2.09	6.33	1.64	5.47	2.27	5.10	0.001	**A = B = C > D**
Hyperactivity	3.14	2.20	2.82	1.63	2.98	2.19	2.67	2.05	0.51	0.68	------
Conduct Problems	5.30	2.02	4.84	1.74	3.79	2.03	2.86	2.24	14.85	0.001	**A = B > C > D**
Peer Problems	4.80	1.76	3.65	1.79	3.27	1.71	3.67	1.59	7.34	0.001	**A > B = C = D**
Pro-social Behrs	8.00	1.86	8.03	1.64	7.88	1.77	8.20	1.76	0.28	.84	---------

As predicted, post hoc contrasts using bonferroni test showed that non-orphaned children whose parents are not known to be infected with HIV/AIDS expressed significantly less emotional problems than children whose parents died of AIDS (t = 1.37, p < .001, d = 0.73), children whose parents died of causes other than AIDS (t = 1.14, p < .003, d = 0.52) and children whose parents are infected or living with HIV/AIDS (t = 0.86, p < . 024, d = 43). However, the differences showed between the latter 3 groups could not reach significance.

Similarly, post hoc comparison indicated that children whose parents died of AIDS reported significantly higher conduct and anti-social problems than children whose parents are infected or living with HIV/AIDS (t = 1.508, p < .001, d = 0.75) and non-orphaned children whose parents are not known to be infected with HIV/AIDS (t = 2.437, p < .001, d = 1.144) but not children whose parents died of causes other than AIDS (t = 0.46, p = n. s.). Subsequently, children whose parents died of causes other than AIDS in turn were significantly higher on conduct problems than children whose parents are infected or living with HIV/AIDS (t = 1.05, p < .01) whilst the latter scored higher than non orphaned children (t = 0.93, p < .05).

In addition, none of the paired multiple post hoc comparisons between groups of children whose parents died of causes other than AIDS, children living with parents infected with HIV/AIDS, and non-orphaned children whose parents are not known to be infected with HIV/AIDS on peer problems reached significance. However, children whose parents died of AIDS were significantly higher on peer problems than children whose parents died of causes other than AIDS (t = 1.153, p < .01, d = 0.65), children whose parents are infected with HIV/AIDS (t = 1.529, p < .001, d = 0.88) and non-orphaned children whose parents are not known to be infected with HIV/AIDS (t = 1.114, p < .01, d = 0.67)

Finally, on total difficulties (sum of emotional, conduct, hyperactivity and peer problems), it showed that children whose parents died of AIDS but not children whose parents died of causes other than AIDS and children whose parents are infected with HIV/AIDS, scored significantly higher than non-orphaned children whose parents are not known to be infected with HIV/AIDS.

### Caseness Definition of Psychological Disorders

Table 2 shows classifications of participants as normal, borderline and abnormal on risks of psychological disorders presented separately for each of the four groups using the standard SDQ cut-off points. A close look at the total SDQ scores showed that 20% and 31.5% of the entire participants fell within the caseness definition for abnormal and borderline respectively. With respect to total problems 22% of children whose parents died of AIDS and 23% of children whose parents are infected with HIV/AIDS respectively fell within abnormal level for psychological disorders compared to 18% for both non-orphaned children whose parents are not known to be infected with HIV/AIDS and children whose parents died of causes other than AIDS. Generally prevalence of abnormal scores was so pronounced for depression and anxiety, measured as emotional problems; 52% for children whose parents died of AIDS and 40% in the entire sample.

**Table 2:** Psychological disorders classification (caseness) for all 4 groups based on SDQ cut-off points

	Groups				
	Orphans of AIDS n = 50	Orphans of other causes (n = 51)	Children living with HIV/AIDs Parents(n = 48)	Non-orphans (n = 48)	Total (n = 200)
**Total Difficulties:**					
Normal	97(49%)	16 (32%)	21 (41%)	29 (60%)	31 (61%)
Borderline	63 (32%)	23 (46%)	21 (41%)	8 (17%)	11 (21%)
Abnormal	40 (20%)	11 (22%)	9 (18%)	11 (23%)	9 (18%)
**Emotional Problems:**					
Normal	62 (31%)	8 (16%)	14 (28%)	11 (23%)	29 (57%)
Borderline	58 (29%)	16 (32%)	15 (29%)	19 (40%)	8 (16%)
Abnormal	80 (40%)	26 (52%)	22 (43%)	18 (37%)	14 (27%)
**Conduct Problems:**					
Normal	132 (66%)	36 (72%)	33 (64%)	29 (60%)	34 (67%)
Borderline	27 (14%)	6 (12%)	8 (16%)	8 (17%)	5 (10%)
Abnormal	41 (21%)	8 (16%)	10 (20%)	11 (23%)	12 (23%)
**Hyperactivity:**					
Normal	178 (89%)	41 (81%)	50 (98%)	43 (90%)	44 (86%)
Borderline	14 (7%)	6 (12%)	1 (2%)	2 (4%)	5 (10%)
Abnorml	8 (4%)	3 (6%)	0 (0%)	3 (6%)	2 (4%)
**Peer Problems:**					
Normal	81 (41%)	10 (20%)	22 (43%)	28 (58%)	21(41%)
Borderline	82 (41%)	22 (44%)	22 (43%)	14 (29%)	24 (47%)
Abnormal	37 (19%)	18 (36%)	7 (14%)	6 (13%)	6 (12%)
**Prosocial Behaviours:**					
Normal	184 (92%)	46 (92%)	47 (92%)	44 (92%)	47 (92%)
Borderline	9 (4.5%)	3 (6%)	2 (4%)	2 (4%)	2 (4%)
Abnormal	7 (3.5%)	1 (2%)	2 (4%)	2 (4%)	2 (4%)

## Discussion

The current results extend the literature on the psychological health of children affected by HIV/AIDS by identifying some interesting findings among groups of children whose parents died of AIDS, children whose parents died of causes other than AIDS, children living with parents infected with HIV/AIDS, and non-orphaned children whose parents are not known to be infected with HIV/AIDS. Considering emotional problems, taken together, children living with parents infected with HIV/AIDS, and both children whose parents died of AIDS and children whose parents died of causes other than AIDS, showed similarly higher symptoms of depression and anxiety than non-orphaned children whose parents are not known to be infected with HIV/AIDS. Summarized differently, indications are that "orphanhood" and parental HIV/AIDS are associated with increased internalizing problems in children. Although this contrasts with other studies [9], the finding is consistent with those who noted that orphans scored higher on adjustment problems such as depression and anxiety than children living with their parents [4,11]. The finding indicates that the impacts that HIV/AIDS have on children's mental health start far before they are made orphan. Research suggests that parental absence and its surrounding trauma are the principal causes of poor childhood adjustment in adolescents [23]. However, in this study, the fact that children living with AIDS parents also reported emotional problems similarly high to both children whose parents died of AIDS and children whose parents died of causes other than AIDS fails to support this claim. I could speculate that the present finding points to family process changes, disruptions to parent-child relationships, non-or-reduced parental guidance, changes in parental moods, and perhaps socioeconomic; conditions that characterize both orphaned families and those with sick parents more accurately could account for higher rates of child conduct problems than the mere parental absence hypothesis [24]. Approximately 37 - 52% of both orphaned groups and children living with parents infected with HIV/AIDS met the criteria for emotional disorders when the caseness cut-off points were considered for risks for abnormality.

The study also found that children whose parents died of AIDS showed more peer problems than all other children. This supports previous and growing evidence which suggest that orphanhood by AIDS is significantly associated with increased peer relationship problems [11,25,26]. Perhaps in Ghana, the stigma and discrimination that people infected with HIV/AIDS face are passed on to their children after the former's death, making their struggle for survival precarious. Stigma is a powerful tool of social control which is often used to marginalize, isolate and make others coil up. AIDS orphaned children may form their own self-schemata based on their daily stigmatized social interactions which then influence multiple aspects of their lives, and results in problems such as the loneliness they reported. Most of these children reported that they have just one or no friends at all and expressed concerns that their peers generally do not like them. The majority of the AIDS orphaned children also noted that they get on well with older people rather than their peers. It is reasoned that it is cumulative effects of HIV/AIDS related stigma and discriminations that underline the higher scores of peer problems in AIDS orphaned children over children living with AIDS parents.

The self-reported levels of hyperactivity among the groups could not reach significance. This is consistent with earlier works that found that children orphaned by AIDS and non-orphaned children did not differ on hyperactivity [9]. Other studies, however, found higher hyperactivity and concentration difficulties among orphaned children [4,27]. In this study, there was approximately only 4% prevalence of abnormal hyperactivity symptoms in the entire sample studied. Compared with a British community sample where abnormal rates of 19.5% and 9.9% were reported for boys and girls respectively the present finding on hyperactivity is encouraging [28].

The relationship between parental death and HIV/AIDS illness on children's conduct problems was mixed, but similar to observations found on the emotional problems. Both orphaned groups scored significantly higher on conduct problems than children whose parents are infected with HIV/AIDS and non-orphaned children whose parents are not known to be infected with HIV/AIDS. However, children living with parents infected with HIV/AIDS expressed significantly higher conduct problems than non-orphaned children whose parents are not known to be infected with HIV/AIDS. Some researcher noted that the possibility of psychopathic behaviours due to children raised without supervision was the greatest fear that relates to the unknown psychosocial effects of orphanhood in Africa [4]. But others questioned this claim, and some failed to find any such evidence [9,29]. However, the current finding yields support for increased self-reported conduct problems among orphans in general and children living with parents infected with HIV/AIDS. The orphaned children reported fighting frequently, stealing, often accused of cheating, and more quickly lost their temper. They were more likely to engage in violent behaviours and develop antisocial attitudes.

### Limitations and Strengths of the study

The general response rate of participants was very high. However, appropriate informants for orphaned children were not readily accessible. Furthermore, time constraints led to reliance on children's self - reports and no other informants recruited. The study recruited small samples of children and no data was collected on how long children were made orphaned. The present findings should also be understood in the context that several demographic characteristics such as the family socioeconomic background, place of residence and prior parent-child relationships were not controlled for. Finally, practitioners should be careful when drawing conclusions about clinical levels of distress reported as the SDQ cut-offs used in the present study have been determined on the basis of research with children in Western Europe and the USA, and that these norms have not been validated in Ghana. However, the strengths of the study include the fact that this is the first study to examine the psychological wellbeing of HIV/AIDS related vulnerable children in Ghana. A further main strength is that the study is the first in Africa and elsewhere that systematically compared groups of children whose parents died of AIDS, children whose parents died of causes other than AIDS, children whose parents are infected/living with HIV/AIDS, and non-orphaned children whose parents are not known to be infected with HIV/AIDS on their psychological outcomes in a single design. Finally, the study used a well established emotional and behavioural measure (Strength and Difficulties Questionnaire) with strong psychometric properties validated for several African countries [21].

## Conclusion

The main finding from the study is that orphaned children and children living with parents infected with HIV/AIDS are at heightened risk for psychological disorders, and in this study, exhibited signs of conduct, peer and emotional problems. The main implication is therefore the need to recognize the increased risk of emotional and behavioural disturbance in these children. Efforts by government agencies and NGO's working with HIV/AIDS affected children should focus on proposals to address these problems in both orphaned children and children living with parents infected with HIV/AIDS. Orphans and children living parents infected with HIV/AIDS are a growing population and so it is urgent that their short and long term psychological needs are cared for. Parallel to this and in the context of the limitations of the study, researchers must see the findings as generated hypotheses rather than conclusions. Further studies are needed to empirically examine larger populations to clarify the specifics of parental HIV/AIDS status or death on children's mental health. Such future studies must engage multiple informants including caregivers, parents, teachers and other immediate family relatives to generate data to be triangulated. It is well acknowledged that children report their own internal states with certainty but are frequently not reliable on their behaviours and by contrast, adults are reliable observers of children's behaviours but frequently underestimate children's internal distress. Future research should also compare orphaned children and foster children on their psychological wellbeing as fostering is not an uncommon practice in Africa.

## Competing interests

The author declares that they have no competing interests.

## Authors' contributions

The author designed the study, carried out the fieldwork and statistical analyses for an MPhil thesis under the supervision of Prof John-Arne Skolkekken. The author drafted this write up alone.
